# Enhancing Gingival Phenotype With Vestibuloplasty and Free Gingival Graft: Improving Maintenance of Regular Oral Hygiene

**DOI:** 10.7759/cureus.23642

**Published:** 2022-03-30

**Authors:** Anootpal Gogoi

**Affiliations:** 1 Periodontology, Regional Dental College, Guwahati, IND

**Keywords:** oral bleeding, periodontal plastic surgery, pocket depth, oral health, recession, oral hygiene, clark’s technique, biotype, free gingival graft, vestibuloplasty

## Abstract

This case report describes Clark’s technique of vestibuloplasty to treat shallow vestibule and, in addition, the use of free gingival autograft to augment attached gingiva to treat Miller’s recession. Vestibuloplasty is performed to deepen a shallow vestibule. Different vestibuloplasty techniques are used to deepen the shallow vestibule by modifying the soft tissue attachment. A 29-year-old male presented to the Department of Periodontics and Oral Implantology, Regional Dental College, Guwahati, India with the chief complaint of bleeding from the lower anterior along with the gingival recession. Maintenance of regular oral hygiene was an added hindrance. The combined technique of vestibuloplasty and use of free gingival graft was performed to achieve dual benefits of increasing the vestibular depth and attainment of a thick gingival phenotype.

## Introduction

A radiant smile results from a healthy body and emotional well-being. The overall smile aesthetics is governed by many factors, such as gingival tissues, form, and position of the teeth [1]. Periodontal plastic surgery plays an important role in enhancing the overall aesthetics. Periodontal plastic surgery, apart from being used as an aesthetic procedure, is also used for improving the maintenance of regular oral hygiene [2]. One such common problem is the shallow vestibule. A shallow vestibule hinders the maintenance of oral hygiene, which leads to incomplete removal of plaque deposits [2]. Sometimes the muscular traction provided by the vestibule can be a causative agent for the development of gingival recession. The presence of a labial frenal attachment can be an additive factor leading to further gingival recession. The depth of the vestibule is measured from the coronal part of the attached gingiva to the muco-buccal fold [3]. Vestibuloplasty is performed to deepen a shallow vestibule. Different vestibuloplasty techniques are used to deepen the shallow vestibule by modifying the soft tissue attachment. This case report describes Clark’s technique of vestibuloplasty [4] to treat the shallow vestibule and, in addition, the use of free gingival autograft to augment attached gingiva to treat Miller’s recession.

## Case presentation

**Figure 1:**
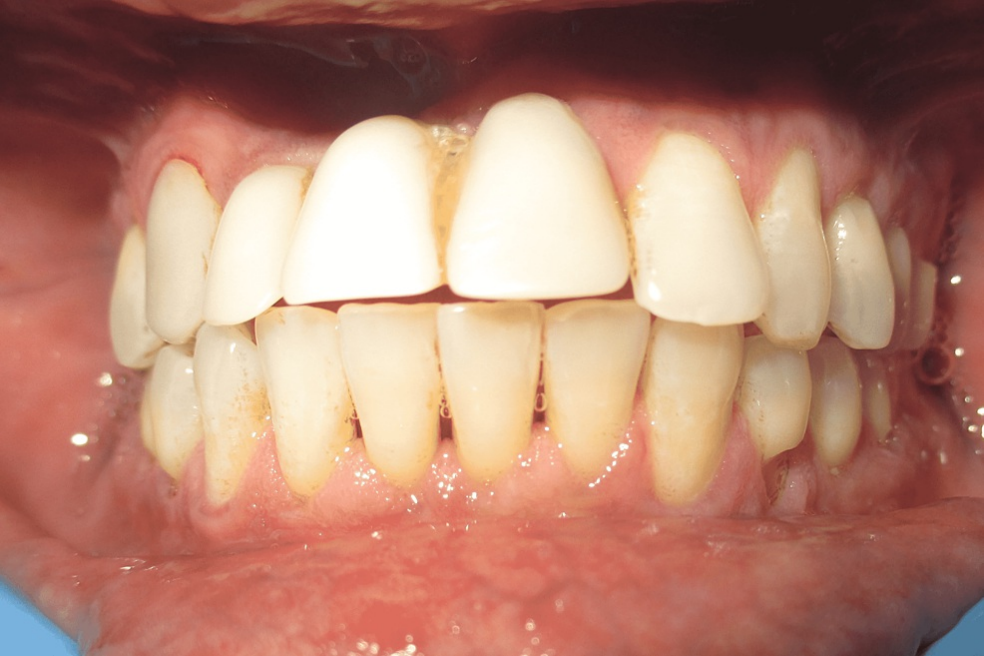
Pre-operative photograph

**Figure 2:**
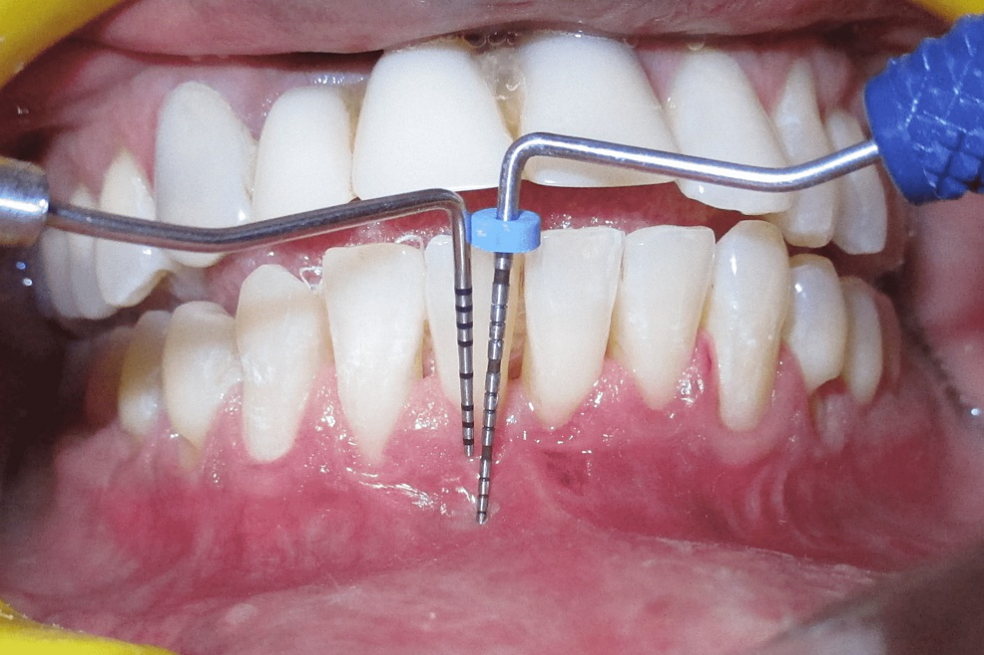
Pre-operative vestibular depth

A 29-year-old male presented to the Department of Periodontics and Oral Implantology, Regional Dental College, Guwahati, India with the chief complaint of bleeding from lower anterior and gingival recession. The patient also had a complaint of not being able to maintain routine oral hygiene due to a shallow vestibule, which prevents the movement of the toothbrush. Following routine examination, it was found that the patient had no previous dental history and was not having any tobacco chewing habits. The patient had a shallow vestibule and the tension test was positive in relation to the lower anterior. Bleeding on probing was present along with gingival recession (Miller’s Class I) in relation to 41, 42, and 31. The patient was suggested vestibuloplasty for treatment of the shallow vestibule along with free gingival autograft to augment the thin attached gingiva following phase I therapy, which included full mouth scaling and gingival curettage in the lower anterior. Pre-operative images are shown in Figure 1 and Figure 2.

**Figure 3:**
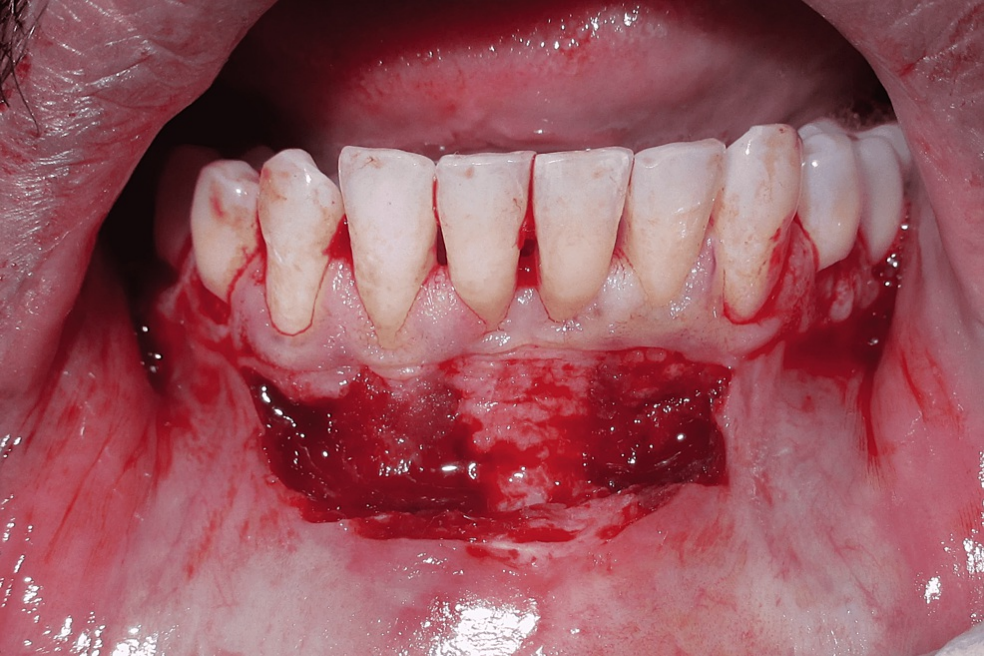
Vestibuloplasty incisions and reflection

Gingival curettage was followed specifically for the lower anterior to treat gingivitis, which occurred due to recurrent deposits of plaque as the patient was not able to maintain his routine oral hygiene. For vestibuloplasty, a horizontal incision was placed at mucogingival junction with respect to 43,42,41,31,32, which was followed by supraperiosteal dissection up to the desired vestibular depth. The mucosa on the labial side was then undermined. The vestibuloplasty incisions are shown in Figure 3.

**Figure 4:**
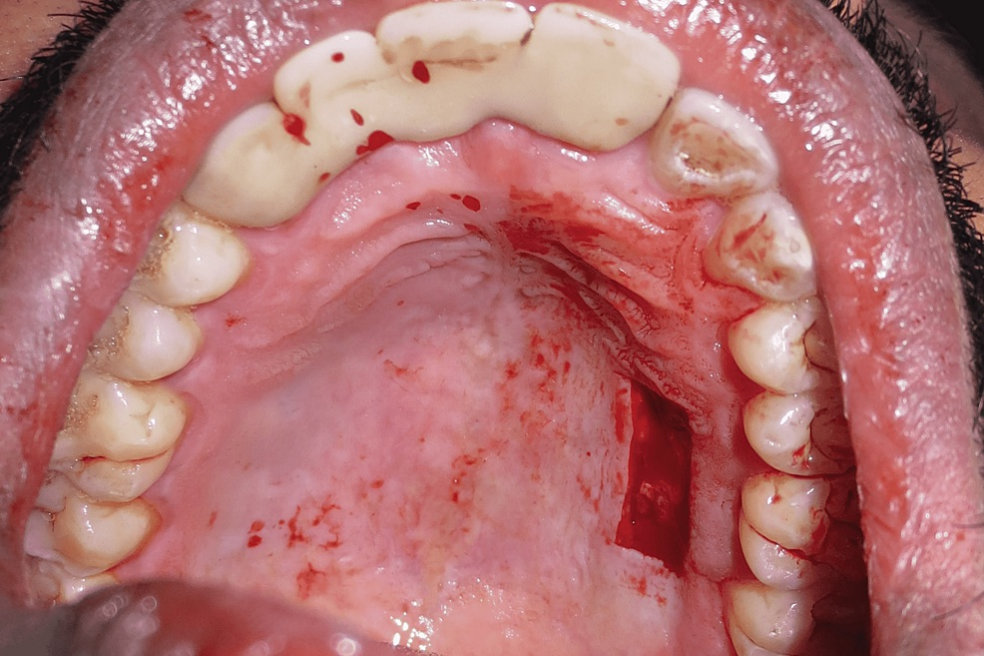
Free gingival graft harvested from the palate

A free gingival autograft was harvested from the palate (Figure 4), the primary purpose of which was to achieve more attached gingiva and to obtain a thicker gingival phenotype.

**Figure 5:**
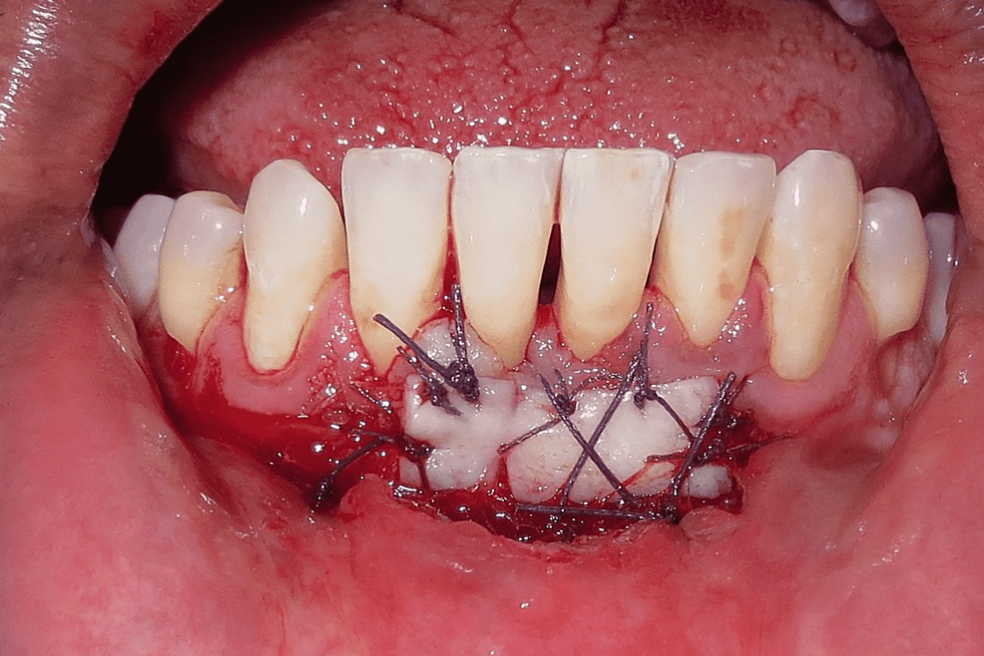
Free gingival autograft stabilized using 5-0 Vicryl

The free gingival autograft was stabilized using a 5-0 resorbable Vicryl suture (Ethicon, Inc., Raritan, New Jersey, United States) (Figure 5) and the undermined area of the mucosa was also sutured at the desired level of the vestibule. Sling sutures were used to stabilize the free gingival graft whereas the mucosa was sutured back by using simple interrupted suture at the desired level of the vestibule.

**Figure 6:**
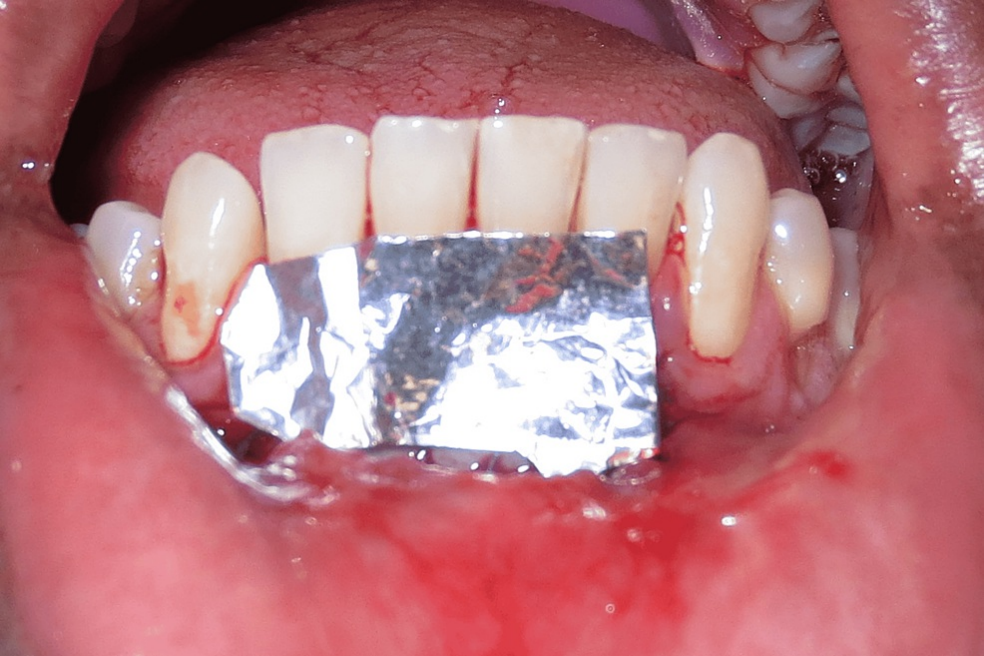
Placement of sterilized tin foil

**Figure 7:**
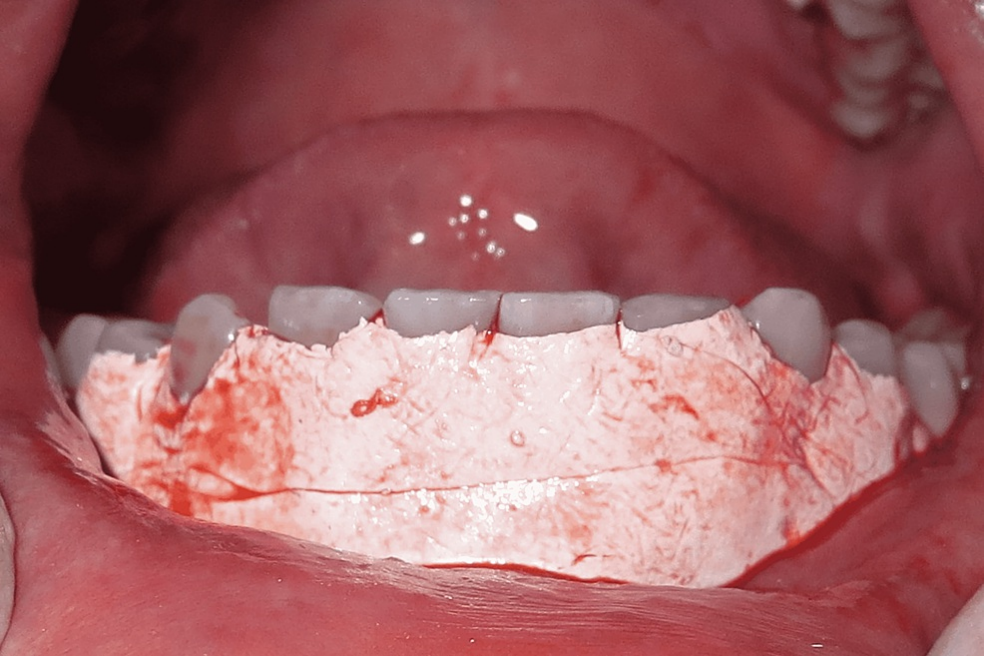
Placement of COE-PAK™ COE-PAK™, GC Corporation, Tokyo, Japan

 A sterilized tin foil is placed to prevent the adherence of COE-PAK™ (GC Corporation, Tokyo, Japan) to the surgical site (Figure 6, Figure 7). COE-PAK acts as a physical barrier protecting the wound from the oral environment.

**Figure 8:**
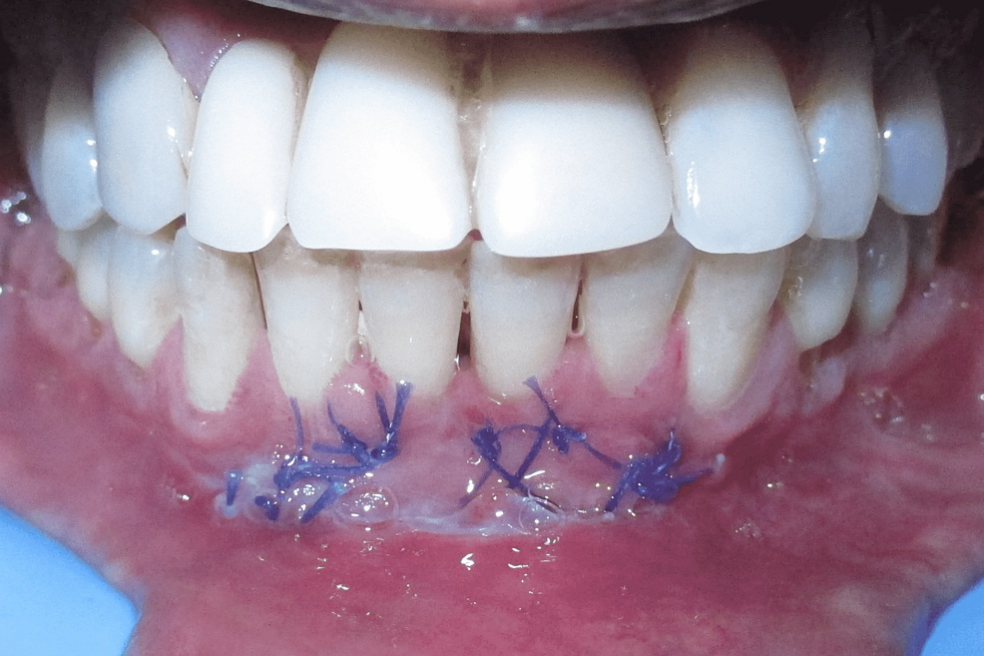
Post-operative evaluation after two weeks

**Figure 9:**
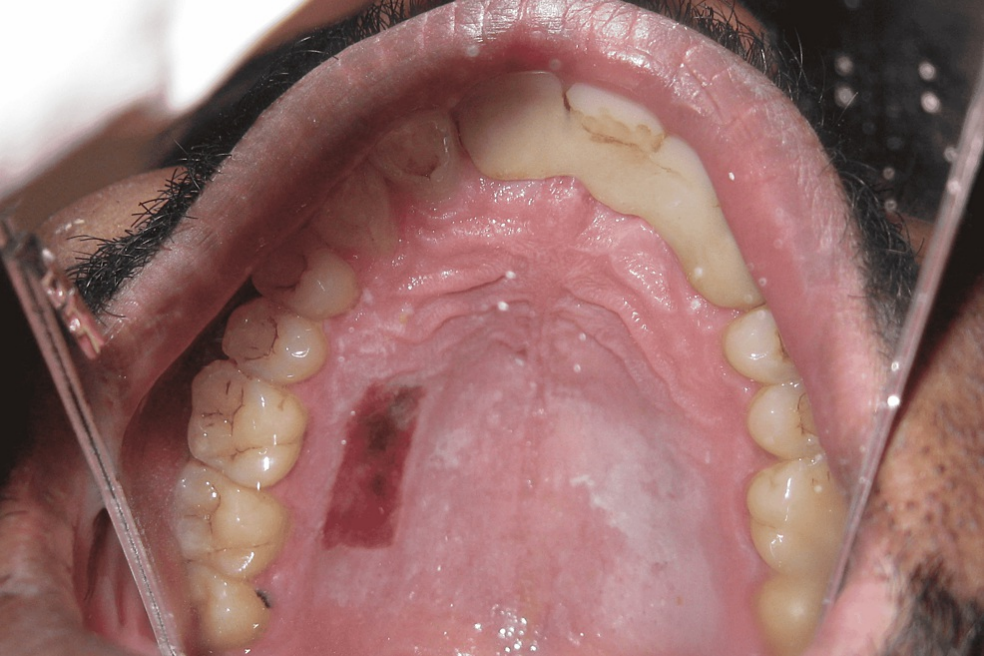
Post-operative evaluation after two weeks (donor site)

The Coe-Pack was removed after 14 days. A post-operative evaluation was done after two weeks (Figure 8, Figure 9).

**Figure 10:**
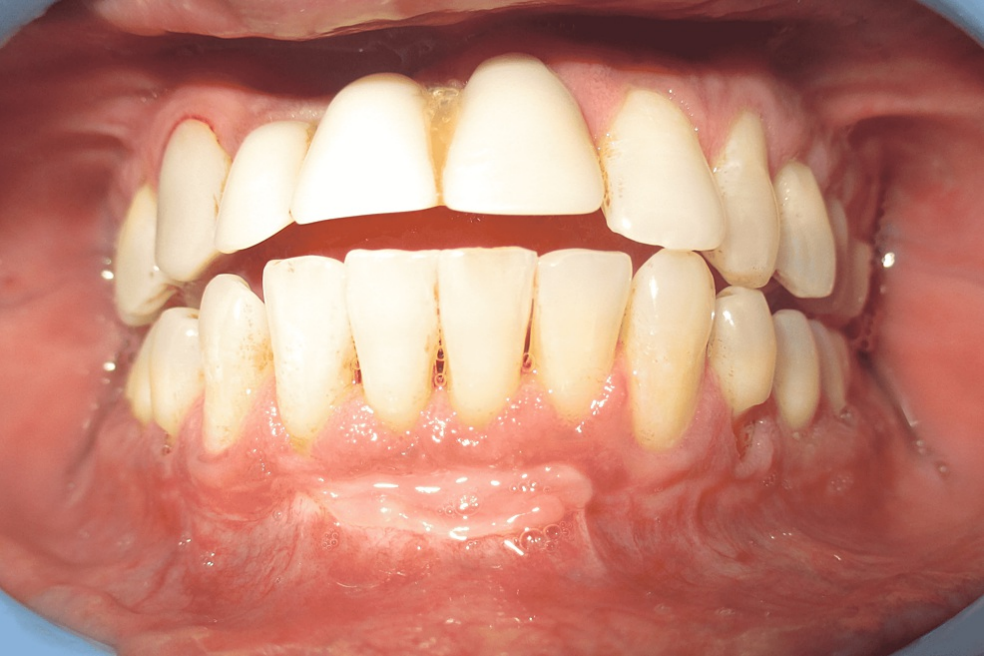
Post-operative evaluation at three months

**Figure 11:**
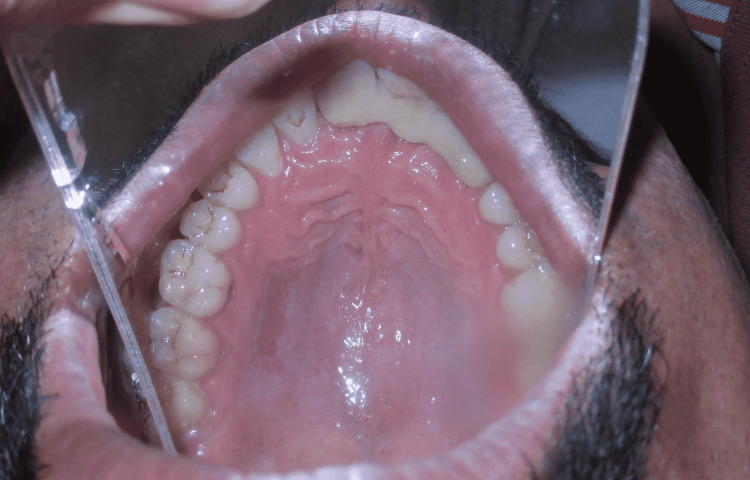
Post-operative evaluation at three months (donor site)

A post-operative evaluation was done again after three months (Figure 10, Figure 11).

## Discussion

Periodontal plastic surgery techniques that were earlier included in the definition of mucogingival surgery are widening of the attached gingiva, deepening of the shallow vestibule, and resection of an aberrant frenum. Deepening of shallow vestibule by vestibuloplasty is a widely-performed technique [5]. Achieving the widening of the attached gingiva and augmenting the thin gingival phenotype along with obtaining a deeper vestibule for maintaining routine oral hygiene is the primary aim of this case. The main reason for having a shallow vestibule is ridge resorption following the extraction of tooth. Other factors relating to shallow vestibule can be firm muscle pull/insertion by positive frenal attachment [6]. This case presents as a gingival recession, which is because of the frenal pull in the lower anterior. Also, the patient had a thin gingival phenotype and a shallow vestibule. Both can lead to the accumulation of plaque in the lower anterior and the possibility of having further recession in the lower anterior also increases [6].

Vestibuloplasty techniques can be broadly classified as mucosal advancement vestibuloplasty, secondary epithelization vestibuloplasty, and grafting vestibuloplasty (Table 1) [7].

**Table 1:** Vestibuloplasty techniques

Techniques	Sub-classification
Mucosal advancement vestibuloplasty	Closed submucosal vestibuloplasty, open submucosal vestibuloplasty (Obwegeser technique )
Secondary epithelization vestibuloplasty	Kazanjian's technique, Godwin's method, Lipswitch method, Clark's method
Grafting vestibuloplasty	Mucosal graft, skin graft

Mucosal advancement vestibuloplasty depends on the height of the bone and the extent of free mobile mucosa along with the vestibule so that adequate deepening can be achieved [8]. If there is an absence of free mobile mucosa along with the vestibule, the secondary epithelization technique has a better prognosis [9 ]. Kazanjian’s technique (1924) was the foremost technique described in the literature for vestibuloplasty. The disadvantage associated with Kazanjian’s technique is extensive scarring of the lip. This scarring is mainly because of the labial flap, which is pedicled off the alveolar process and used to cover the alveolar bone side. The labial surface heals by secondary epithelization. Clark (1953) recommended flap be pedicled off the lip and the raw area be left on the alveolar side rather than the labial side [10]. The disadvantage of Clark’s technique is its unpredictability in maintaining the vestibule depth. Clark's technique, which heals by secondary epithelization mechanism, can cause reinsertion of detached muscles to their pre­operative levels, causing a relapse in vestibular depth [9]. In this case, besides Clark’s technique of vestibuloplasty, a free gingival graft harvested from the palate was used to augment the thin gingival phenotype and prevent further recession. Details of literature on free gingival graft are given in Table 2.

**Table 2:** Literature review for free gingival graft

Year	Author	Conclusion
2022	Kayaalti-Yüksek et al. [10]	It was concluded that although both techniques were effective, Gingival unit graft can be a convenient method for treatment of bilateral recession type 1 (RT1) gingival recessions with inadequate keratinized tissue width (KTW) and (or) shallow vestibule depth.
2022	Lin et al. [11]	Free gingival grafting was found to be a predictable treatment approach for augmentation of keratinized mucosa width (KMW) around implants in the posterior region after the fabrication of prostheses as long as grafts of sufficient size were placed. Stable outcomes were shown in the study participants during the follow-up period of up to three years.
2021	Edranov et al. [12]	The results indicate activation of mesenchymal stem cells in the area of localization of the graft and differentiating osteoblasts. The observed osteoinductive effect of free gingival graft is associated with its participation in reorganization in mesenchymal stem cells and induction of morphogenetic molecules.
2021	Huang et al. [13]	Free gingival graft could result in a greater increase of keratinized mucosa width (KMW) than xenogeneic collagen matrix (XCM). Both could increase KMW, maintain peri-implant health, and attain comparable aesthetic outcomes. The use of XCM was associated with reduced operation time
2020	Ku et al. [14]	Vestibuloplasty with a free gingival graft using titanium mesh could be achieved with an acceptable amount of keratinized gingiva and an appropriate vestibular depth around the dental implant.
2020	Tarasenko et al. [15]	Despite the limitations of the study, it was found that free gingival graft (FGG) was the most effective technique to augment the amount of keratinized attached mucosa (KM) in sites of implant placement. The use of a collagen matrix could be a viable alternative to diminish the intervention's impact on patients' postoperative quality of life.
2019	Kinaia et al. [16]	The use of partly de-epithelialized free gingival graft can be used to enhance keratinized gingiva (KG) for lingual recession with adequate root coverage.
2018	Falabella et al. [17]	Follow-up indicated that the use of the free gingival graft was effective in increasing keratinized tissue and in covering the recession in a satisfactory manner.

The periodontal phenotype has been proposed to replace the term gingival biotype [18]. The term periodontal phenotype, first proposed by Müller and Eger, includes the three-dimensional gingival volume and thickness of the buccal/facial bone plate [19]. The free gingival graft will help in the prevention of further recession [20]. Lesser invasive procedures like the use of collagen matrix-like Geistlich Mucograft® (Geistlich Pharma AG, Wolhusen, Switzerland) and Mucoderm® (botiss medical AG, Berlin, Germany) can be used as an alternative to autogenous grafts like free gingival grafts for the treatment of such similar scenarios [13].

## Conclusions

This case report shows the successful outcome of vestibuloplasty in the anterior mandible. The combined technique of vestibuloplasty and the use of free gingival graft have dual benefits of increasing the vestibular depth and attainment of a thick gingival phenotype. The deeper vestibule will make routine oral hygiene possible and prevent the accumulation of plaque. 

## References

[1] Garber DA, Salama MA (1996). The aesthetic smile: diagnosis and treatment. Periodontol 2000.

[2] Zucchelli G, Mounssif I (2015). Periodontal plastic surgery. Periodontol 2000.

[3] Ward VJ (1976). A technique of measurement of the depth of the vestibular fornix in the mandibular anterior region. J Periodontol.

[4] Peterson LJ (2011). Peterson’s Principles of Oral and Maxillofacial Surgery, Volume I, Third Edition. https://www.google.co.in/books/edition/Peterson_s_Principles_of_Oral_and_Maxill/Gxo8AwAAQBAJ?hl=en&gbpv=1&dq=inauthor:%22Larry+J.+Peterson%22&printsec=frontcover.

[5] Ozgul O, Senses F, Er N (2015). Efficacy of platelet rich fibrin in the reduction of the pain and swelling after impacted third molar surgery: randomized multicenter split-mouth clinical trial. Head Face Med.

[6] Natarajan S, Banu F, Kumar M, Lavu V (2019). Management of shallow vestibule with reduced attached gingiva in fixed prosthetic intervention. Cureus.

[7] CL HB Jr (1953). Deepening of labial sulcus by mucosal flap advancement. J Oral Surg (Chic).

[8] Chari H, Shaik KV (2016). Preprosthetic surgery: review of literature. IJSS Case Reports.

[9] Hillerup S (1980). Healing reactions of relapse in secondary epithelization vestibuloplasty on dog mandibles. Int J Oral Surg.

[10] Kayaalti-Yüksek S, Yaprak E (2022). The comparison of the efficacy of gingival unit graft with connective tissue graft in recession defect coverage: a randomized split-mouth clinical trial. Clin Oral Investig.

[11] Lin IP, Chang CC, Tu CC, Lai CL, Su FY (2022). Efficacy of free gingival grafting to augment keratinized mucosa around dental implants in posterior regions after restorative procedures: a retrospective clinical study. J Prosthet Dent.

[12] Edranov SS, Matveeva NY, Kalinichenko SG (2021). On-bone fixation of free gingival graft induces an osteoinductive effect in human alveolar bone. Bull Exp Biol Med.

[13] Huang JP, Liu JM, Wu YM (2021). Clinical evaluation of xenogeneic collagen matrix versus free gingival grafts for keratinized mucosa augmentation around dental implants: A randomized controlled clinical trial. J Clin Periodontol.

[14] Ku JK, Leem DH (2020). Retrospective case series analysis of vestibuloplasty with free gingival graft and titanium mesh around dental implant. J Korean Assoc Oral Maxillofac Surg.

[15] Tarasenko S, Ashurko I, Taschieri S, Repina S, Esaya N A, Corbella S (2020). Comparative analysis of methods to increase ﻿the amount of keratinized mucosa before stage-two surgery: a randomized controlled study. Quintessence Int.

[16] Kinaia BM, Kazerani S, Hsu YT, Neely AL (2019). Partly deepithelialized free gingival graft for treatment of lingual recession. Clin Adv Periodontics.

[17] Falabella ME, Alvarenga FF, Segalla KB, Adão SR, Silva DG, Silva-Boghossian CM (2018). Treatment of gingival recession in 2 surgical stages: free gingival graft plus coronally positioned flap. Gen Dent.

[18] Jepsen S, Caton JG, Albandar JM (2018). Periodontal manifestations of systemic diseases and developmental and acquired conditions: Consensus report of workgroup 3 of the 2017 World Workshop on the Classification of Periodontal and Peri-Implant Diseases and Conditions. J Periodontol.

[19] Müller HP, Eger T (1997). Gingival phenotypes in young male adults. J Clin Periodontol.

[20] Rateitschak KH, Egli U, Fringeli G (1979). Recession: a 4-year longitudinal study after free gingival grafts. J Clin Periodontol.

